# Engineering Individual Oxygen Vacancies: Domain-Wall Conductivity and Controllable Topological Solitons

**DOI:** 10.1021/acsnano.1c03623

**Published:** 2021-08-06

**Authors:** Hemaprabha Elangovan, Maya Barzilay, Jiawei Huang, Shi Liu, Shai Cohen, Yachin Ivry

**Affiliations:** † Department of Materials Science and Engineering, Technion−Israel Institute of Technology, Haifa 3200003, Israel; ‡ Solid State Institute, Technion−Israel Institute of Technology, Haifa 3200003, Israel; § School of Science, Westlake University, Hangzhou, Zhejiang 310024, China; ∥ Institute of Natural Sciences, Westlake Institute for Advanced Study, Hangzhou, Zhejiang 310024, China; □ Key Laboratory for Quantum Materials of Zhejiang Province, Hangzhou, Zhejiang 310024, China; # Nuclear Research Centre-Negev, Beer-Sheva 84190, Israel

**Keywords:** individual oxygen vacancy, linear quadrupole, domain-wall charging, topological soliton, domain-wall conductivity, differential phase contrast, electron-beam tomography

## Abstract

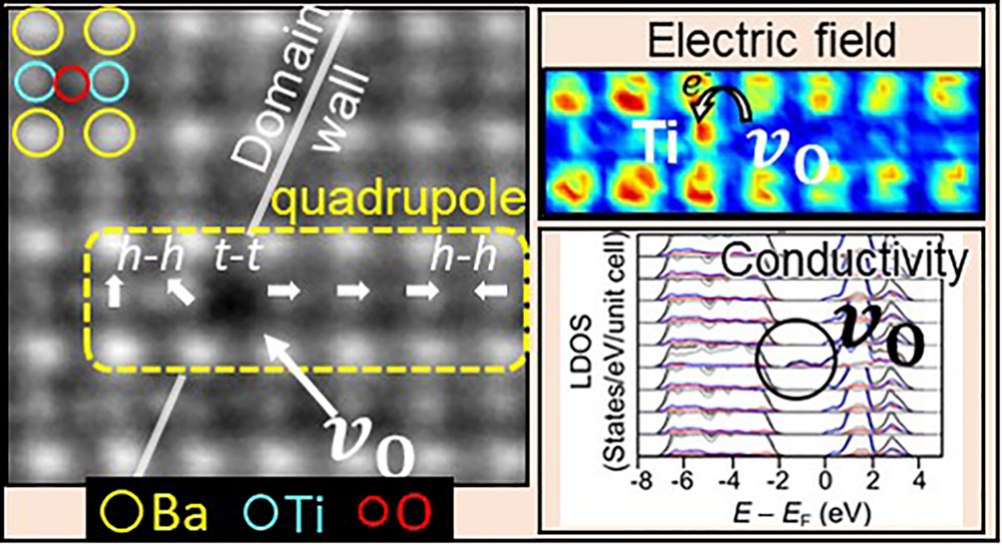

Nanoscale devices that utilize oxygen vacancies in two-dimensional metal-oxide structures garner much attention due to conductive, magnetic, and even superconductive functionalities they exhibit. Ferroelectric domain walls have been a prominent recent example because they serve as a hub for topological defects and hence are attractive for next-generation data technologies. However, owing to the light weight of oxygen atoms and localized effects of their vacancies, the atomic-scale electrical and mechanical influence of individual oxygen vacancies has remained elusive. Here, stable individual oxygen vacancies were engineered *in situ* at domain walls of seminal titanate perovskite ferroics. The atomic-scale electric-field, charge, dipole-moment, and strain distribution around these vacancies were characterized by combining advanced transmission electron microscopy and first-principle methodologies. The engineered vacancies were used to form quasi-linear quadrupole topological defects. Significant intraband states were found in the unit cell of the engineered vacancies, proposing a meaningful domain-wall conductivity for miniaturized data-storage applications. Reduction of the Ti ion as well as enhanced charging and electric-field concentration were demonstrated near the vacancy. A 3–5% tensile strain was observed at the immediate surrounding unit cells of the vacancies. Engineering individual oxygen vacancies and topological solitons thus offers a platform for predetermining both atomic-scale and global functional properties of device miniaturization in metal oxides.

## Introduction

Oxygen vacancies in metal oxides are the basis for modern nanoscale electronics because they can modify the functional properties significantly without affecting the structure and the mechanical properties of these materials.^1^ Examples include ferromagnetism in LaCoO_3_,^2^ conductive filament formation in metal-oxide–metal structures,^3^ enhanced catalytic activity in SrCoO_3−δ_,^4,5^ and ferroelectric-superconductivity coexistence in Sr_1–*x*_Ca_*x*_TiO_3−δ_,^6^ even though these materials are insulating and nonmagnetic in their stoichiometric form. Recent focus is put on 2D structures. The interface between two metal oxides, such as LaAlO_3_/SrTiO_3_, is a common example.^7−9^ A prominent platform for such 2D structures is ferroelectric domain walls, which separate neighboring regions with different dipole-moment and polarization orientations.^10^ Oxygen vacancies have been found lately serving as a hub for topological defects, such as vortices and skyrmions as well as altering significantly the properties of the encapsulating high-dielectric parent material,^11−14^ for instance by introducing 2D magnetism (Hf_0.5_Zr_0.5_O_2_^15^) and 2D superconductivity (WO_*x*_^16^) at the domain walls. Substantial effort has been given though to domain-wall conductivity in large-bandgap ferroelectrics, vastly because domain walls are movable by will with external electric fields, giving rise to miniaturized memristive cells.^17−19^ While the success of presenting domain-wall conductivity in traditional perovskite ferroelectrics, such as BaTiO_3_^20^ and Pb(Zr_0.2_Ti_0.8_)O_3_, has remained limited,^21−23^ attention has been given mainly to BiFeO_3_,^24−27^ ErMnO_3_,^28,29^ and LiNbO_3_,^30−32^ which show high and reproducible conductivity.

The origin of oxygen-vacancy-based functionality is at the atomic scale. Yet, observing, controlling, and even modeling individual vacancies have remained a major challenge. Macroscopic-scale effects of oxygen vacancies are observed readily as an averaged behavior with spectroscopic methods.^33−37^ Changes in the material electronic band structure and charge-carrier concentration, both contributing to conductivity, have been observed as coupled with an increase in oxygen-vacancy concentration.^38,39^ At the mesoscopic scale, enhanced conductivity is frequently observed at walls that separate head-to-head (*h–h*) or tail-to-tail (*t–t*) polarization domains with scanning probe microscopy.^40^ Theoretical^41^ and modeling^42^ analyses of these structures predict that such charged *h–h* (*t–t*) domain walls repel (attract) oxygen vacancies, which in turn help stabilize these charged domain walls globally. Recent atomic-scale scanning transmission electron microscopy (STEM) studies of bismuth ferrite demonstrated clusters of charged point defects at domain walls.^43,44^ Initially, indirect observations attributed these defects to oxygen vacancies.^45,46^ However, very lately, electron energy loss spectroscopy (EELS) studies determined that charged defects at the domain walls are most likely due to bismuth vacancies, questioning the existence and role of oxygen vacancies at the domain walls.^47^ A major reason for the unresolved role of individual oxygen vacancies is that modeling point defects at domain walls within a collectively-interacting dipole matrix is complex. Likewise, observing these light atoms is a nontrivial task, and spotting individual vacant sites is even a larger hurdle, especially at the domain wall.^48−50^ That is, direct observations of the basic individual oxygen vacancies and the electric field around it as well as the ability to manipulate these basic building blocks have remained elusive, limiting the development of advanced metal-oxide-based nanoscale devices.

Here, we combined various atomic-scale imaging and *in situ* manipulation techniques together with density functional theory (DFT) modeling to demonstrate intentional formation of oxygen vacancies at domain walls in the seminal ferroelectric perovskite BaTiO_3_. Complementary calculations expanded the work to other perovskite ferroelectrics and not only supported the stability of oxygen vacancies at the domain walls, but also illustrated that they give rise to intragap states, which are significant for conductance. Individual vacancies were found to be inducing mechanical strain and electrical charging at a distance of up to two unit cells, which is much smaller than previous predictions.^51−53^ Careful analysis of the dipole-moment, electric-charge, electric-field, and strain distribution around the vacancies showed a quasi-linear quadrupole that comprises a pair of *h–h* and *t–t* dipole moments. The contribution of this nonconventional topological structure to the local electric-field distribution at the domain wall was demonstrated independently.

## Results and Discussion

Single-crystal BaTiO_3_ 50 nm crystallites were used, providing strain-free bulk-like behavior, while yet allowing sufficient electron transparency to aid the TEM analyses. Recently, it has been shown that ferroic domain walls can be formed, moved, and switched contactless in such materials *in situ* during atomic-scale TEM imaging.^54,55^ Here, we used this method to form the domain-wall structure, which served as a template for the oxygen vacancies. The guiding hypothesis was that oxygen vacancies are formed at a window dosage above the value that forms the domain walls and before the material is damaged. Simultaneous STEM-based differential phase contrast (DPC), integrated differential phase contrast (iDPC), high-angle annular dark field (HAADF), and EELS were used to characterize the atomic-scale chemical-element, strain, charge, and electric-field distribution, exact atomic location of the ions, and the ions’ oxidation state, respectively (see Methods for further explanation about these imaging modes).

**Figure 1:**
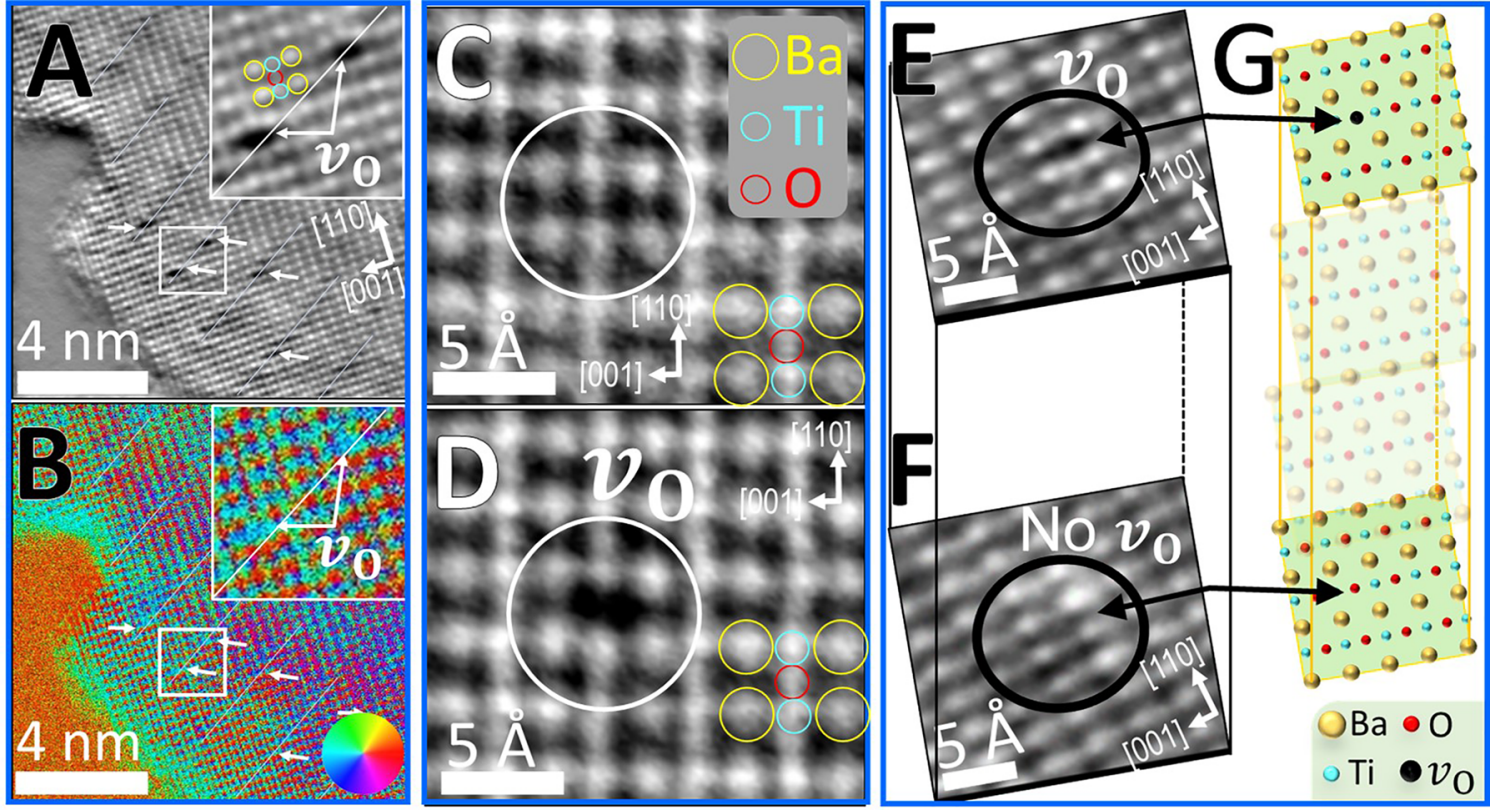
Formation and direct observation of oxygen vacancies at 90° domain walls in BaTiO_3_. (A) Atomic structure (iDPC image) and (B) the simultaneously imaged electric-field distribution at the same area (DPC micrograph) of artificially formed oxygen vacancies at 90° domain walls in the BaTiO_3_ crystallite. Representative oxygen vacancies (*v*_O_) are highlighted in the insets. Striped domain walls (DW) appear as a change in contrast in (A) and (B) and the simultaneously imaged HAADF signal (Figure S1). Color wheel in (B) represents the orientation (color) and intensity (hue) of the electric-field displacement vector. (C) Atomic structure of an unperturbed BaTiO_3_ crystallite. (D) The same area after intentional oxygen-vacancy formation (exposure to 66 nA/nm^2^*in situ*). (E) Oxygen vacancy at a certain depth within a BaTiO_3_ crystallite. (F) Changing the focal depth to a plane that is located 2 ± 1 nm exactly below (E) shows that all the atomic sites are occupied and no oxygen vacancies exist. (G) Schematic depth profiling of the oxygen-vacancy localization as was observed in (E) and (F). Larger scale images of (A)–(D) are given in Figure S1.

Figure 1A shows the atomic structure (iDPC) of an area in which artificial domains (striped 90° domains) were formed *in situ*. Oxygen vacancies appear clearly as points of “missing” atoms with black contrast. The vacancies are located along the domain walls that appear not only in Figure 1A but also in the simultaneously imaged electric-field distribution in that area (DPC, Figure 1B) and HAADF image (Figure S1). A detailed discussion regarding the oxygen vacancy within the 3D matrix geometry is presented in Figure S2.

To show controllability of the vacancies, first, the atomic structure of an unperturbed crystal was mapped (Figure 1C). Figure 1D shows the same area after successful intentional *in situ* formation of an oxygen vacancy within a domain wall (details about the domain-wall formation procedure are provided in ref (54)).

Localization of the oxygen vacancies was demonstrated with the aid of high sensitivity to focal depth of the iDPC method.^56^Figure 1E,F shows that the vacant sites are localized at a certain plane within the material. That is, although the vacancy is noticeable in Figure 1E, by changing carefully the focal depth, Figure 1F shows that the same site at the same area albeit at a plane that is placed only 2 ± 1 nm below the plane in Figure 1F is now occupied by an oxygen atom and no vacancy was detected (see schematics in Figure 1G). Large-scale micrographs of Figure 1A–F are given in Figures S1 and S2.

**Figure 2:**
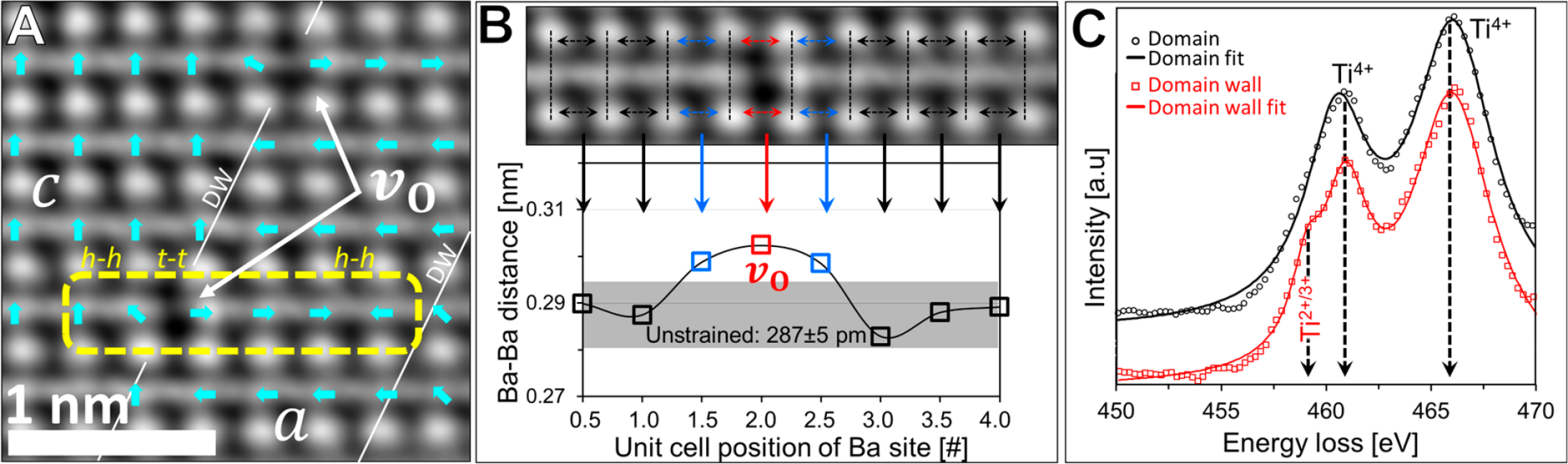
Atomic-scale mapping of the charge-strain distribution near an oxygen vacancy site at a domain wall. (A) iDPC image of oxygen vacancies at a domain wall. A quasi-linear quadrupole (*h–h t–t h–h*) dipole-moment arrangement around a vacancy is marked in yellow dashed lines. (B) The Ba–Ba distance distribution near the vacancy demonstrates 5% tensile strain at the vacant site and 3.5% at the adjacent site. The following unit cells are unstrained. (C) EELS spectra from domains and a domain wall near the Ti-L_23_ peak, showing a reduced state of titanium ions at the domain walls (Figure S5).^59−62^

Figure 2A shows the dipole-moment distribution across the domain wall based on the Ti ion displacement (the domain wall is identified also with a simple contrast analysis of the larger scale HAADF, DPC, and iDPC signals; see Figure S3). Two oxygen vacancies were observed here along the wall, and the dipole-moment distribution around them was identical. In both cases, the dipole moments were aligned *t–t* at the site with the missing oxygen atom, accompanied by an *h–h* alignment at the immediate neighboring unit cells from each side. Beyond the distance of already one unit cell, the dipole moments returned to their unperturbed *h*–*t* orientation. Previous studies proposed that charged vertices of dipole moments (non-*h–t*) are accompanied by strain release.^39^Figure 2B shows the strain distribution across the unit cells with charged vertices (top), which was evaluated based on the distance between neighboring barium atoms. First, a 287 ± 5 pm Ba–Ba distance was measured for unit cells with no oxygen vacancies around them, which is in agreement with the literature.^57,58^ In comparison to this value, Figure 2B (bottom) shows 5% expansion for the vacant site (302 ± 3 pm) and 3.5% expansion for the immediate neighboring sites (297 ± 2 pm). The strain was relaxed already at the next unit cells, complying with the dipole moment distribution. To show the reproducibility of this behavior, data were collected for three oxygen-vacancy sites and over 50 unperturbed unit cells as demonstrated in Figure S4.

To further confirm the charging at the vacant site, atomic-resolution EELS measurements were done across the domain wall. Figures 2C and S5 reveal that the oxidation state of the titanium ion changes from Ti^4+^ within the domains to a lower oxidation state at the domain wall,^59−62^ where the oxygen vacancies are. Using the Kröger–Vink notation, the *v*_O_ formation can be summarized: O_O_^×^ → *v*_O_^••^ + 2*e*′ + ^1^/_2_O_2_, so that the Ti ion transfers due to the vacancy from +4 to +2 to maintain charge neutrality, whereas it can also transfer to +3 to allow charging.

Summing up the dipole-moment distribution around the oxygen vacancy shows an *h–h/t–t/h–h* structure. Typically, in perovskite ferroelectrics, dipole moments arrange in *h*–*t* to maintain charge neutrality, whereas charged domain walls comprise an *h*–*h* or *t*–*t* structure. In both cases, although the charge distribution is asymmetric, the dipole-moment distribution itself is centrosymmetric. Thus, in these cases, the system can be described by means of dipole moments only. Nevertheless, in the current scenario of an *h–h/t–t/h–h* structure, the dipole moment distribution is not centrosymmetric, requiring a higher-order analysis of the system in terms of the multipole expansion. That is, this alternating dipole moment organization represents an individual unit of a quadrupole. The alternating dipole moments orient nearly on one common line, with the exception that the dipole moments near one of the *h–h* vertices has an additional orthogonal component (within the image plane). Thus, this structure is a quasi-linear quadrupole. Traditionally, dipole moments dominate the behavior of ferroelectrics and other dielectric materials. Higher orders of the multipole expansion, such as quadrupoles, are considered typically as negligible for the macroscopic behavior of the material because their contribution is significant only at the short-range.^63^ Nevertheless, in the context of domain walls, and in particular oxygen vacancies and other point defects at domain walls, short-range interactions become prominent.^64^

**Figure 3:**
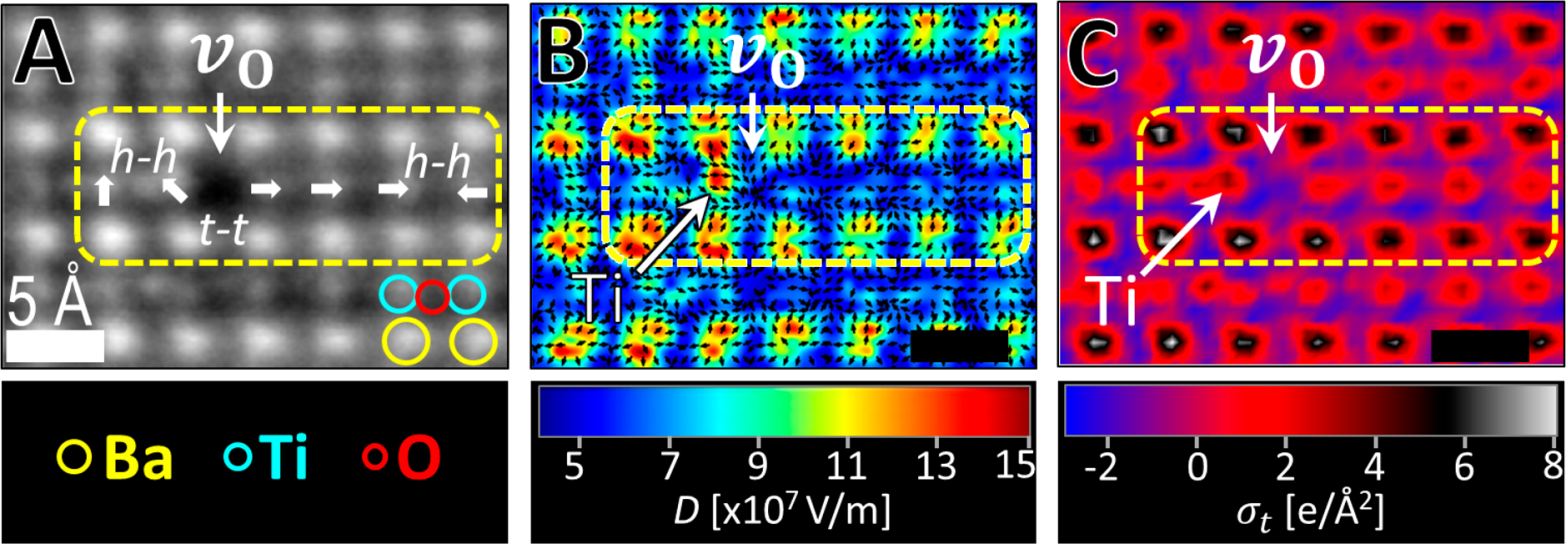
Enhanced displacement field and charge density near an oxygen vacancy. (A) iDPC image near an oxygen vacancy (a quasi-linear quadrupole is highlighted). (B) Displacement-field (*D*) vector map representing the orientation of the field, overlapped on the strength of the field. The field enhancement is seen at the *h–h* dipole near the vacant site. (C) Calculated (eq 1) charge density around the oxygen vacancy, showing only background charge at the vacant site as well as a strong charge of a larger ionic radius for the nearby Ti ion (both are highlighted). All scale bars are 5 Å. Larger scale images are given in Figure S6.

The short-range electric-field distribution around oxygen vacancies was therefore examined at the subatomic scale by means of DPC. Figure 3A shows the atomic structure of a BaTiO_3_ crystallite with an oxygen vacancy at the domain wall. The simultaneous iDPC mapping in Figure 3B shows the absence of an electric field at the vacancy site. Likewise, a locally enhanced electric field is observed around the titanium ion that is at the adjacent *h–h* charged vertex (note that to reduce the noise level, 5 × 5 pixel averaging was done^45,65^). This local electric-field enhancement is typical for such a quasi-linear quadrupole.^66^ Following Gauss’s law, the local charge distribution can be extracted from the electric field; however, this should be done carefully. Mostly, the local field that is mapped with DPC is the electric field, *E*, which corresponds to the free charge density, ρ_f_. Because in ferroelectrics, bound charge (ρ_b_) and the resultant polarization dominate ρ_f_ and *E*, the entire displacement field (*D*) must be taken into account^67^ (see additional technical discussion in the SI). That is, the charge density around the oxygen vacancy can be extracted from the divergence of the DPC mapping (Figure 3B):
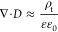
1where ρ_t_ is the total bound and free charge density, ε_0_ is the vacuum permittivity, and ε is the dielectric constant of the material. DPC micrographs are two-dimensional. Thus, integration of eq 1 over the sample thickness allows us to extract the areal charge density (σ_t_).

Figure 3C shows the charge distribution around an oxygen vacancy. Dominant charge density was observed at the Ba sites with respect to Ti and oxygen sites both near and far away from the vacancy. Only background charge is observed at the vacant site. The charge at the nearby Ti site (at the region with the higher displacement field) was distributed over a large area with respect to the other titanium ions. Because the atomic radius of a titanium ion increases with decreasing oxidation states,^68^ the latter is in agreement with the above EELS results. Note that the oxidation state of the titanium ion was changed only in the charged *h*–*h* vertex closest to the vacancy. This asymmetric charge distribution is accompanied by an off-axis diagonal displacement of the reduced Ti ion (Figure 3C).

**Figure 4:**
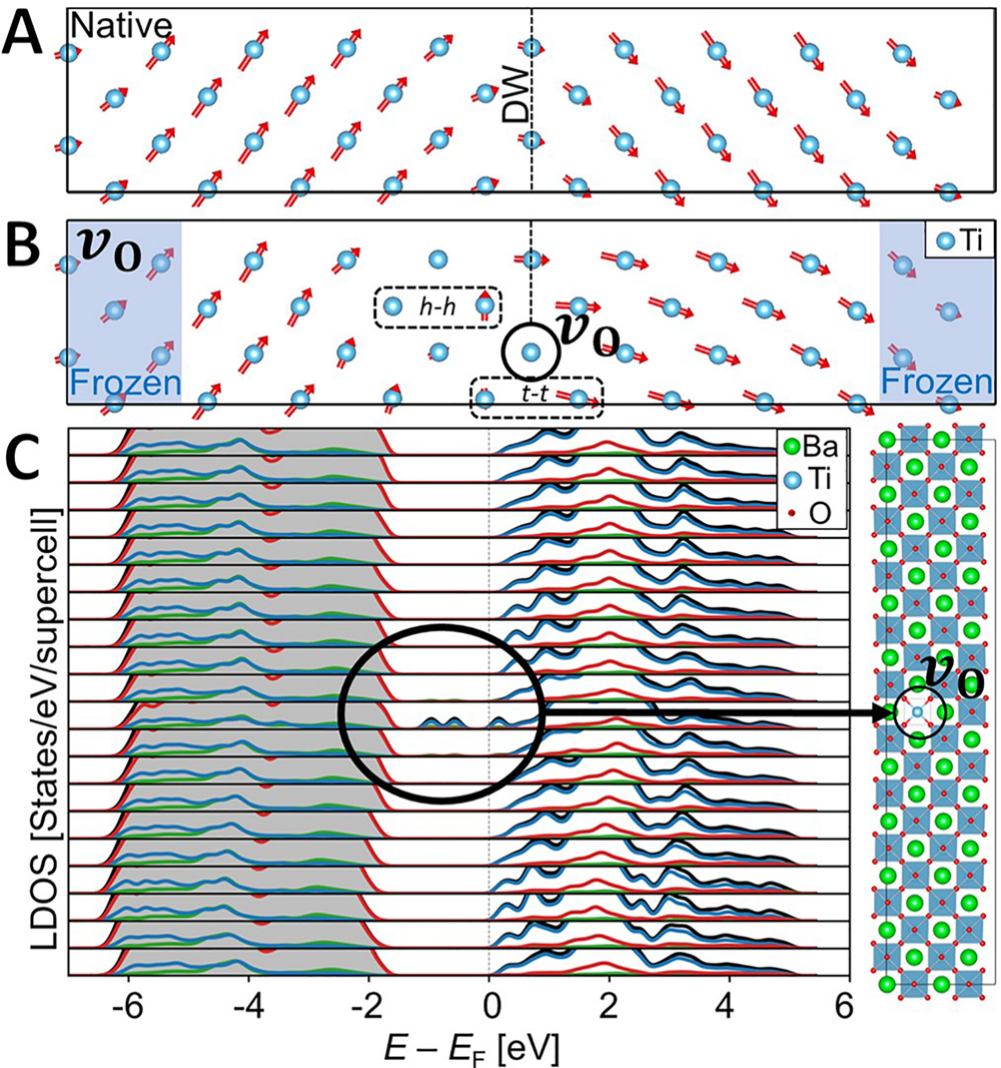
Local structure and projected density of states around an oxygen vacancy computed with DFT. (A) Dipole-moment distribution near a 90° domain wall in BaTiO_3_. (B) Stable oxygen vacancy at the domain wall with induced charging *h–h* and *t–t* vertices. (C) Contribution to the local DOS (left) of individual unit cells near an oxygen vacancy at a 90° domain wall (right). Red, blue, and green curves represent contributions from O, Ti, and Ba atoms, respectively, while the black curve is the total DOS.

There is a strong motivation to understand the effects of oxygen vacancies on the global material behavior of the ferroelectric, in addition to the above characterized local microscopic effects. In particular, there is a technological interest in understanding the effects of oxygen vacancies on domain-wall conductivity. Thus, complementary modeling was done to expand the realm of the above microscopic characterization (please see SI for further details regarding the DFT conditions). Figure 4A shows a DFT modeling of a 90° domain wall in BaTiO_3_ that comprises an oxygen vacancy. We note that modeling a 90° domain wall in the tetragonal phase of BaTiO_3_ with DFT turns out to be a nontrivial task. First, DFT calculations are typically done at 0 K, at which BaTiO_3_ adopts the rhombohedral ground-state phase, whereas a tetragonal structure of BaTiO_3_ is stable only at finite temperatures. Second, the energy of a 90° domain wall in BaTiO_3_ is expected to be even smaller than that of a 180° domain wall (7.84 mJ/m^2^).^69,70^ In comparison, the energies of 180° and 90° walls in PbTiO_3_ are 170 and 90 mJ/m^2^, respectively.^71,72^ Modeling a low-energy interface demands a high accuracy in energy and atomic forces. Moreover, to maintain a reasonable oxygen vacancy concentration at the wall, a relatively large supercell is needed, further increasing the computational cost. These subtle issues likely explain the lack of DFT studies on 90° domain walls in BaTiO_3_ in the literature.^73,74^ Here we followed a protocol developed to study highly unstable charged domain walls.^42^ A large 10√2 × 2√2 × 1 supercell of 200 atoms was adopted to minimize artificial interactions between a defect and its mirror images. The supercell was divided into three zones: atoms in the two end zones were fixed to the bulk values of the tetragonal phase, while atoms in the middle region were allowed to relax. This allowed for structural optimization of a low-energy 90° wall sandwiched by two bulk tetragonal domains (fixed end zones). First, the optimized structure of defect-free 90° walls was obtained (Figure 4A), and then a neutral oxygen vacancy was introduced at the wall, following a similar protocol to that used in previous studies,^75^ which was located at the center of the supercell (Figure 4B). The local polarization of each unit cell was evaluated by the displacement of the central Ti atom with respect to its surrounding oxygen octahedron.

The DFT simulations agree with the experimental data in several important aspects. First, the simulations show an asymmetric dipolar distribution of the charged *t–t* and *h–h* vertices near the oxygen vacancy. Likewise, oxygen vacancies are found stable also at domain walls that are as dense as 2 nm periodicity (in agreement with Figure 1A). Finally, Figure 4B shows that the dipole moments relax already at the distance of *ca*. 2 unit cells from the vacancy. Also, note that the experimentally observed reduction of the Ti ion (Figures 2C and 3C) is consistent with the calculated charge redistribution in the DFT modeling of this neutral-charge system (see Figures S7 and S8).

To examine the effect of oxygen vacancies on the domain-wall conductivity, layer-resolved local density of states (LDOS) was computed. Figure 4C shows the existence of significant intragap states at the vacant site (fewer states exist also at the adjacent unit cells), whereas no midgap states were observed elsewhere. As a comparison, we examined the effects of the oxygen vacancy on the electronic structures of 90° domain walls in PbTiO_3_. Three different types of oxygen vacancies at the wall were studied, all leading to meaningful intragap states at the interface (Figure S9). This suggests that the defect levels due to oxygen vacancies are promising for enhanced conductivity.

## Conclusions

Most studies propose that domain-wall conductivity arises from a coherent *h–h* or *t–t* dipole-moment organization at the domain wall, which in turn can serve as a hub for oxygen vacancies (and other point defects). The above results show that *h–h* and *t–t* can be local effects, giving rise to substantial changes in the local electric-field, charge, mechanical strain, dipole and quadrupole moment distribution at the domain wall, and the oxidation state of nearby ions. It is possible that local effects are more prominent in the case of 90° ferroelastic domain walls than in 180° domain walls because of the lower symmetry of the former. However, we believe that future work is required to examine this hypothesis.

The oxygen-vacancy manipulation presented in this work was obtained with the electron beam at a certain dosage window. Previous works showed that for dosage values less than 4 × 10^–4^ nA/nm^2^, the dominant interaction of the sample and the electron beam is electromagnetic, rather than, say, local heating, resulting in domain-wall formation and switching.^54,55^ Moreover, it has been shown that for dosage values greater than or equal to 270 nA/nm^2^, the electron beam interacts locally with the sample, giving rise to cation escape and even to atomic-scale etching of the material.^59^ In the current work, it was shown that individual oxygen-vacancy engineering is possible at the dosage window between these two values. That is, the electron beam has already formed domain walls, while it induces local charging and isolated point defects, without yet damaging the material. Figure S10 shows the emergence of an oxygen vacancy in a switched domain wall, supporting this conclusion. Note that following the experimental results and the DFT calculations, we expect a similar behavior for the oxygen-vacancy formation also in systems in which the domain walls were formed differently.

A quasi-uniaxial quadrupole that is located at a domain wall that separates between two areas with clear dipole-moment and polarization orientation was found to be conjugated with the engineered oxygen vacancy. This spatially localized change in symmetry is similar to a sudden change in direction in the middle of a spiraling telephone cord, indicating that the quadrupoles in this work serve as a topological soliton. This topological structure is promising for nanoscale electronic technologies and deserves further investigation. Note that the localized strain (mechanical, chemical, and electric) release in the current work stems from the high domain-wall periodicity in comparison to the typical periodicity.^51−53^ Moreover, on many occasions (see Figure 1A for example), the oxygen vacancies appeared in an organized superlattice-like structure, following the domain-wall meta-structure. Consequently, formation of a correlated or even network oxygen-vacancy structure is possible. Utilizing such correlated point defects and topological solitons as an individual entity for technological applications may enhance the functionality of these metal-oxide materials.

The contribution of an atomic-scale topological structure was found to be important not only to local properties but also for macroscopic properties, such as electric conductivity. The above results highlight the significance of local topological and structural effects, including quadrupoles, which have been considered negligible with respect to the macroscopic and device-relevant properties. Likewise, the above oxygen-vacancy engineering methodologies can be implemented for manipulating exotic structures and functional properties of ferroelectrics and other metal-oxide materials.

## Methods

### Material

Commercially available single-crystal BaTiO_3_ nanoparticles of 50 nm size^54,59,76^ were purchased from US Research Nanomaterials, Inc. (99.9% pure). To evenly spread the particles on the TEM grid, the particles were suspended in ethanol and sprayed on the amorphous carbon-coated holey Cu grid using nitrogen gas.

### TEM

The TEM experiments were carried out on an aberration-corrected Titan Themis 80–300 operated at 200 kV. For STEM-DPC experiments, the equipment was set with a dose of 10–250 pA. The oxygen vacancies are formed at a dosage window between 4 × 10^–4^ and 270 nA/nm^2^ (the experiments presented in Figures 1 and 2 involved also higher dosage values). The probe semiconvergence angle of 21 mrad and collecting semiangles of 25–154 mrad were used for the HAADF mode. The DPC images were captured using a segmented (four-quadrant) annular dark-field detector (DF4), with collecting semiangle of 6–34 mrad. To demonstrate the reproducibility of the oxygen-vacancy engineering, >100 oxygen vacancies were formed in eight different experiments that involved eight different crystallites.

A key factor that helped with the characterization is that while the contrast in traditional STEM and HAADF methods is quadratic with the atomic number—decreasing the signal-to-noise ratio of light elements—the DPC methods are sensitive also to lighter elements, such as oxygen.^56^ The contrast in DPC and iDPC can be also more localized at a specific focal plane than conventional STEM imaging methods. Hence, by changing the focal depth, tomography-like characterization of the structural and electric properties was obtained. Moreover, in DPC, the electron beam diversions due to local changes in the electrical and magnetic fields within the sample are detected, allowing us to extract the local electric-field distribution (see detailed discussion in the SI). Lastly, the high accuracy of atom location obtained with iDPC^56^ was used to map the dipole-moment distribution with confidence.

### DFT

All first-principles DFT calculations were performed using QUANTUM ESPRESSO^77^ with generalized gradient approximation of the Perdew–Burke–Ernzerhof for solids (PBEsol) type. Given the large supercell used to model domain walls, we used the GBRV ultrasoft pseudopotential^78^ and a 1 × 1 × 4 Monkhorst–Pack *k*-point grid for structural optimization and a 2 × 2 × 8 *k*-point grid for electronic structure calculation. The plane-wave cutoff is set to 40 Ry, and the charge density cutoff is set to 200 Ry, respectively.^79^

## Supporting Information Available

Large-scale micrographs for images used in the manuscript; TEM-based tomography around individual oxygen vacancy; Ba–Ba distance distribution in a native crystal; spatial domain and domain walls and the corresponding EELS spectra of the entire range that covers the oxidation states of Ba, Ti, and O; DPC-based charge-density calculation; lattice parameters; formation energy of oxygen vacancies at domain walls; charge-density and Bader charge calculation by DFT; electronic band structure calculation for PbTiO_3_ with an oxygen vacancy; oxygen-vacancy formation at an existing domain wall (PDF)
