# Supplementary material for: Engineering Individual Oxygen Vacancies: Domain-Wall Conductivity and Controllable Topological Solitons
Source: ACS Nano. 2021 Aug 6;15(8):13380–8. doi: 10.1021/acsnano.1c03623 (PMC8631733; doi:10.1021/acsnano.1c03623)
Supplement: Supplementary file 1 — nn1c03623_si_001.pdf [file nn1c03623_si_001.pdf]

Supplementary Information for: *"Engineering Individual Oxygen Vacancies: Domain-Wall Conductivity and Controllable Topological Solitons"*

Hemaprabha Elangovan,[†,1,2] Maya Barzilay,[†,1,2] Jiawei Huang,[3,4,5] Shi Liu,[3,4,5] Shai Cohen[6] and Yachin Ivry[1,2,*]

[1] Department of Materials Science and Engineering, Technion – Israel Institute of Technology, Haifa 3200003, Israel.

[2] Solid State Institute, Technion – Israel Institute of Technology, Haifa 3200003, Israel.

[3] School of Science, Westlake University, Hangzhou, Zhejiang 310024, China.

[4] Institute of Natural Sciences, Westlake Institute for Advanced Study, Hangzhou, Zhejiang 310024, China.

[5] Key Laboratory for Quantum Materials of Zhejiang Province, Hangzhou Zhejiang 310024, China.

[6] Nuclear Research Centre-Negev, Beer-Sheva 84190, Israel.

[*]Correspondence to*: ivry@technion.ac.il*

[†] These authors contributed equally to the work.

SI – Table of Contents

Large-scale micrographs S2
Depth profile around an oxygen vacancy S3
Ba-Ba distance in a native $BaTiO_3$ structure S5
EELS S5
DPC-based charge-density calculation S6
Density Functional Theory Modeling S8
  Unit-cell parameters S8
  Energetically favorable formation of v_O at domain walls. S8
  Charge density and Bader Charge S8
  Density of States (DOS) for $PbTiO_3$ S9
  Oxygen-vacancy formation at an existing domain wall S10
References S11

Large-scale micrographs

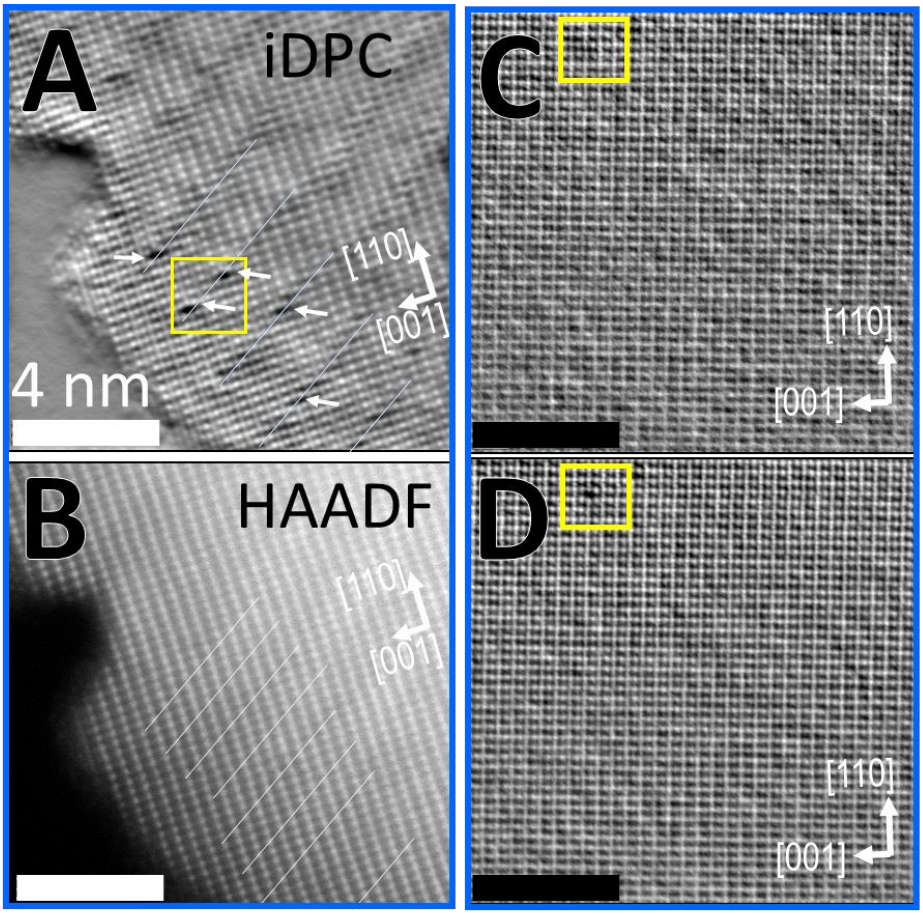

Figure S1| Oxygen vacancy formation at 90° domain walls. Simultaneously imaged (**A**) iDPC; (**B)** HAADF-STEM showing domain walls (highlighted) with oxygen vacancies of the area presented in Figure 1A-B. (**C**) iDPC image of the native structure of $BaTiO_3$, (**D**) iDPC micrograph of the same area as in (C) with an oxygen after an exposure to a dosage of 66 nA/nm^2, vacancy.

Depth profile around an oxygen vacancy

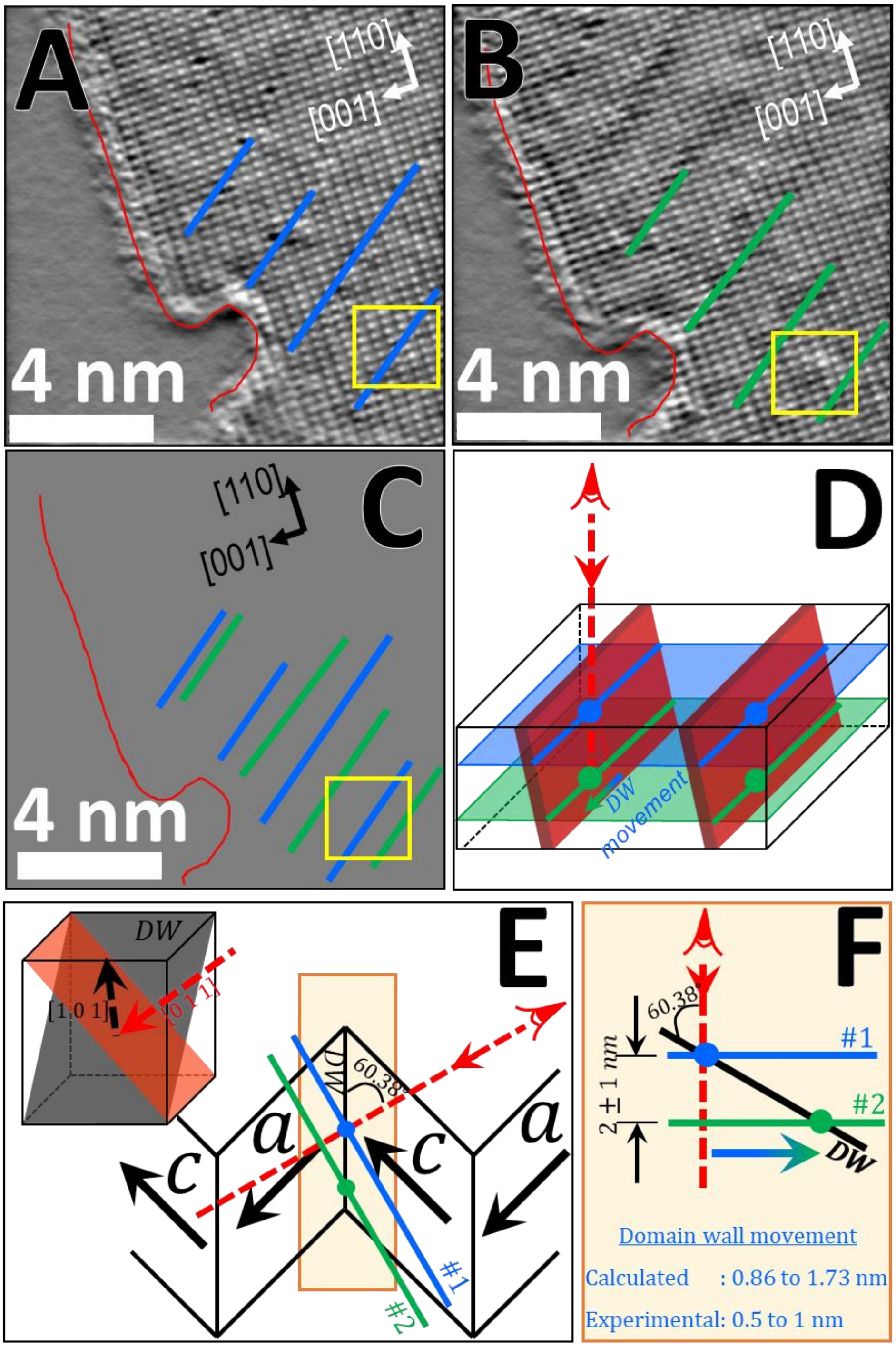

Figure S2| Depth profile around an oxygen vacancy at a 90° domain wall structure. (**A**) iDPC scan of an area that comprises oxygen vacancies and 90° domain walls. (**B**) The same area after changing the focal plane 2 ± 1 nm below (A). (**C**) Schematics of the crystalline with blue and green lines that represent the position of the domain walls as appear in (A) and (B), respectively, showing ~1 nm lateral displacement. (**D**) Schematic explanation of the concept of depth profiling of an area that comprises 90° domain walls and an oxygen vacancy. (**E**) Schematic illustration of the specific geometry as was imaged in the zone axis used in Figure 1. (**F**) Side-view projection (adjusted) of the profiling that is demonstrated in (E).

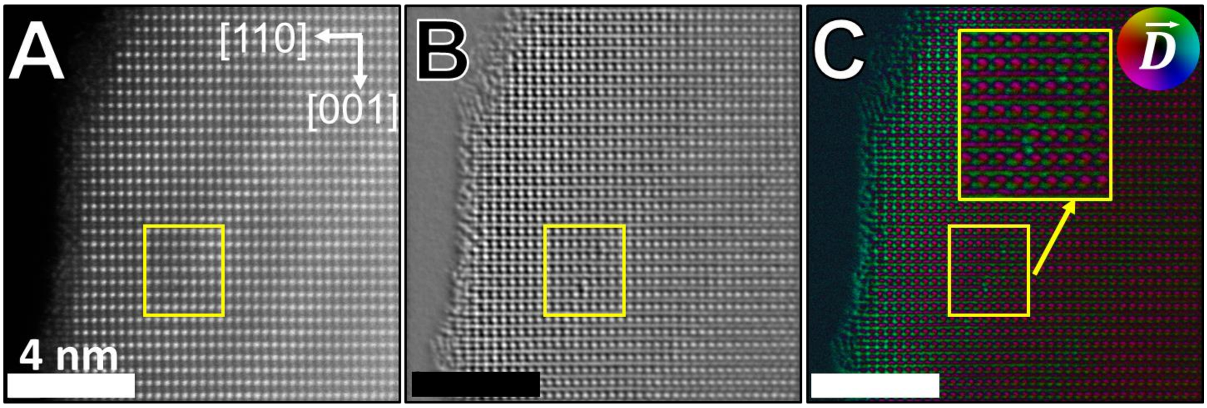

Figure S3| Mechanical strain and dipole-moment distribution around an oxygen vacancy. Simultaneously imaged (**A**) HAADF-STEM; (**B**) iDPC and (**C**) DPC micrographs, showing that two oxygen vacancies appear clearly at the iDPC and DPC images. The dipole distribution and the strain around these vacancies are presented in Figure 2A-B. The area shown in Figure 2A-B is highlighted in (B). Color wheel in (C) represents the orientation (color) and intensity (hue) of the electric-field displacement vector. All scale bars are 4 nm.

Ba-Ba distance in a native $BaTiO_3$ structure

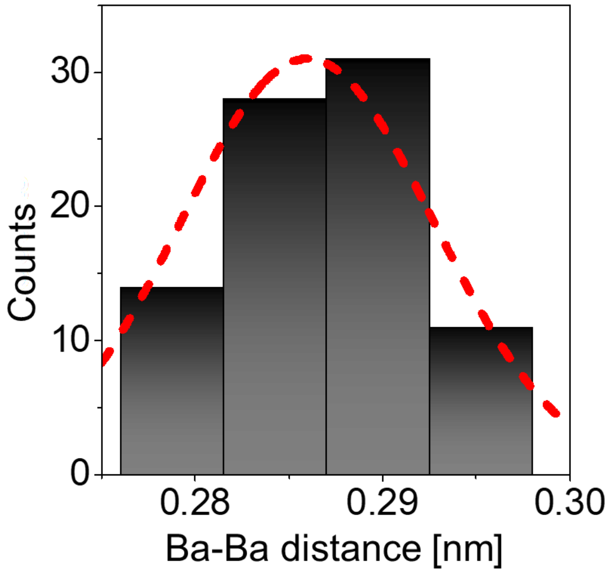

Figure S4| Ba-Ba distance distribution within a pristine structure of $BaTiO_3$. Ba-Ba distance along the [011] direction as was measured for >50 sites that do not contain nearby oxygen vacancies, showing 287±5 pm.

EELS

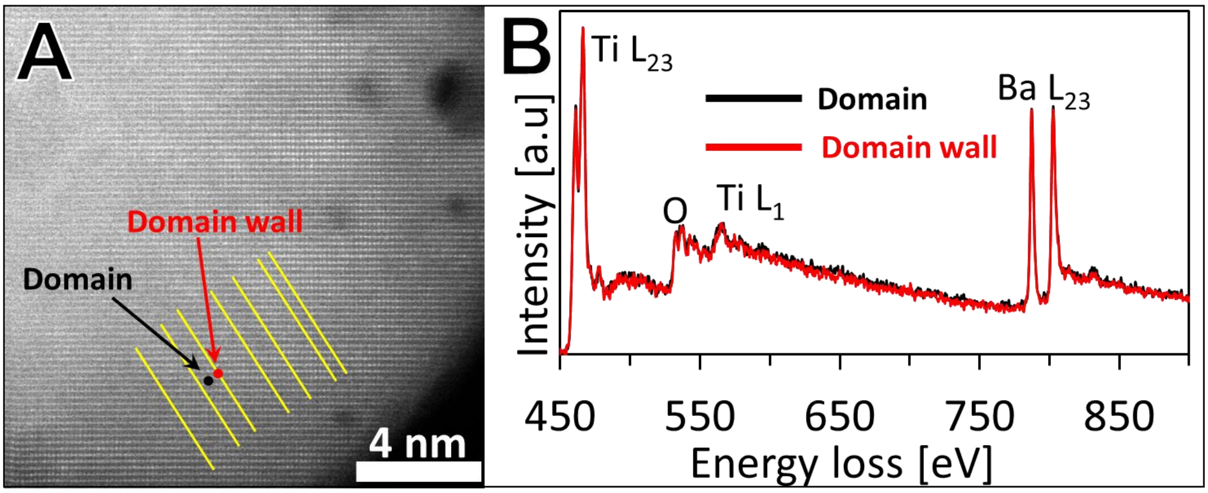

Figure S5| EELS characterization across domain walls. (**A**) An Annular Dark-Field (ADF) micrograph of an area within a $BaTiO_3$ crystallite with high-periodic domains.[1] (**B**) Large-area EELS spectra of data collected from within the domains (black) and the domain walls (red), indicating the energy position of Ba, Ti and O ions. The Ti L_{23} is shown in Figure 2C. Each curve is an average of five different measurements along the line highlighted in (A).

DPC-based charge-density calculation

DPC is a STEM method, at which the electron-beam deflects due to local electromagnetic interactions with the sample, which exerts on it a Lorentz force. In the case of ferroelectrics, the deflection is vastly due to the contribution of bound charges to the displacement field $(\vec{D})$. Four sensitive detectors (A-D) that are organized together in the shape of a ring are awaiting to measure the electron beam after traveling through the sample. The deflection is correlated with the magnitude of $\vec{D}$ and maintains its orientation, allowing spatial mapping of the electromagnetic field within the material, following,

$$|\vec{D}| = \alpha \frac{I_{\mathrm{A-C}}(x,y)\hat{\imath} + I_{\mathrm{B-D}}(x,y)\hat{\jmath}}{t\, I_{\mathrm{sum}}(x,y)} \qquad \text{(S1)},$$

where α is a calibration factor, (x,y) is a coordinate at the sample plane, $I_{\mathrm{i-j}}(x,y)$ is the intensity difference the signal collected at the i and j detector quadrants from the (x,y) coordinate, $I_{\mathrm{sum}}(x,y)$ is the total intensity at the four quadrants for the (x,y) coordinate, *i.e.*, sum of the signals of all the four detectors and t is the sample thickness (~10 nm at the area of interest in this work).

The calibration factor corresponds primarily to the microscope and detector parameters:

$$\alpha = \left(\frac{R^2 - r^2}{R.C}\right)\left(\frac{mv}{e}\right) \qquad \text{(S2)},$$

while R is the radius of the beam size at the detector plane (4 mm) and r represents the inner radius of the ring formed by the detectors (0.7 mm) at a camera length of C (295 mm). e, m, and v are the electron charge and relativistic mass, and velocity, respectively for a given acceleration voltage (here, 200 kV). Micrographs of the individual quadrant detectors that were used to extract the displacement field and charge density distribution in Figure 3 are given in Figure S6.

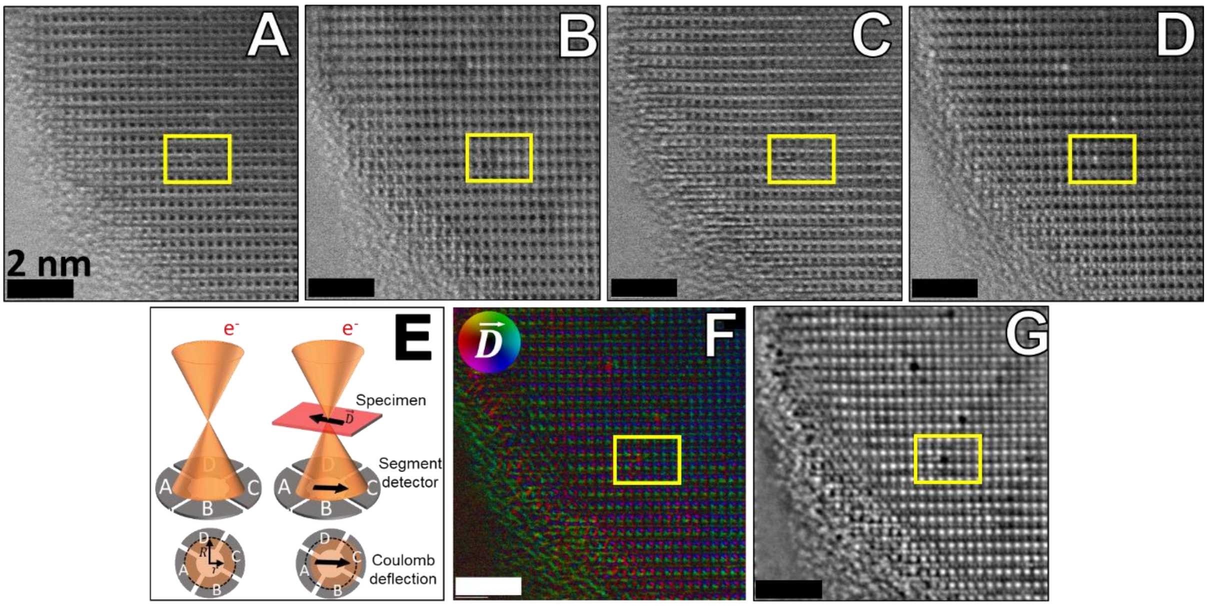

Figure S6| Atomic-scale DPC segment-detector and iDPC mapping. (A-D) Intensity signals collected at the respective A, B, C and D segments of the DPC detector. These signals were used to calculate the field and charge density in Figure 3, following Equations 1 and S2. (**E**) Simplified schematics of the electron beam deflection due to electric-field distribution in the specimen. (**F)** DPC and **(G)** iDPC images extracted from the segment-detectors signals (A-D, 0.5 s integration time) showing oxygen vacancies within domain walls. Yellow boxes denote the region of interest. Color wheel in (F) represents the orientation (color) and intensity (hue) of the electric-field displacement vector. All scale bars are 2 nm.

Density Functional Theory Modeling

Unit-cell parameters

To model a 90° domain wall, we followed the protocol detailed in Reference[2]. The dimensions of the supercell contained two 90° domain walls (one at the center and one at the boundary because of the periodic boundary conditions) are computed directly using PBEsol optimized lattice constants of tetragonal phase of $BaTiO_3$, $a_1 = a_2 = 3.959$ Å and $c = 4.022$ Å. The theoretical c/a ratio for a tetragonal unit cell is 1.016, agreeing well with experimental value of 1.011.[3] We fixed the supercell dimensions to these values, L_x=56.43 Å, L_y = 5.6437 Å, L_z = 3.959 Å, and then fully relaxed the atomic positions.

Energetically favorable formation of v_O at domain walls.

Previous studies have already shown that the oxygen vacancy in the domain wall has lower formation energy compared to that in bulk. For example, He and Vanderbilt showed that vacancies have lower formation energy in the 180° domain wall in tetragonal $PbTiO_3$.[4] Chandrasekaran *et al.* found that both $Fe_{Ti}'' - v_O^{\cdot\cdot}$ and $v_{Pb}'' - v_O^{\cdot\cdot}$ are more stable in 180° domain walls of $PbTiO_3$.[5] More recently, Wang *et al.* calculated the formation energy of oxygen vacancy in the 90° domain wall of $PbTiO_3$ and found the oxygen vacancy is more stable in the domain wall.[6]

Charge density and Bader Charge:

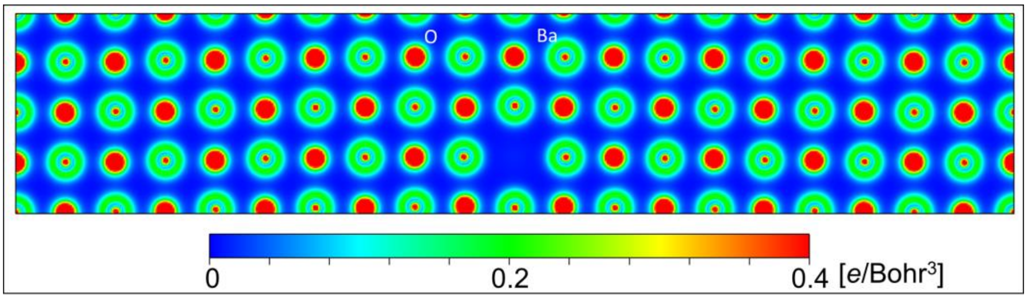

Figure S7| Charge density of $BaTiO_3$ calculated from DFT.

Further, we computed the effective Bader charges for the supercell containing oxygen vacancies in 90° domain walls. The calculated Bader charges for Ba, Ti, and O in the bulk-like region were 1.53*e*, 2.19*e*, and -1.25*e*, respectively, agreeing well with previous reports (*e* is the electron charge).[7] Notably, the Ti atom in proximity to v_O in the domain wall has a much lower effective charge of 1.67*e*, a 24% reduction. A complementary Lowdin population analysis revealed a similar charge reduction for the Ti atom close to v_O in 90° domain walls.

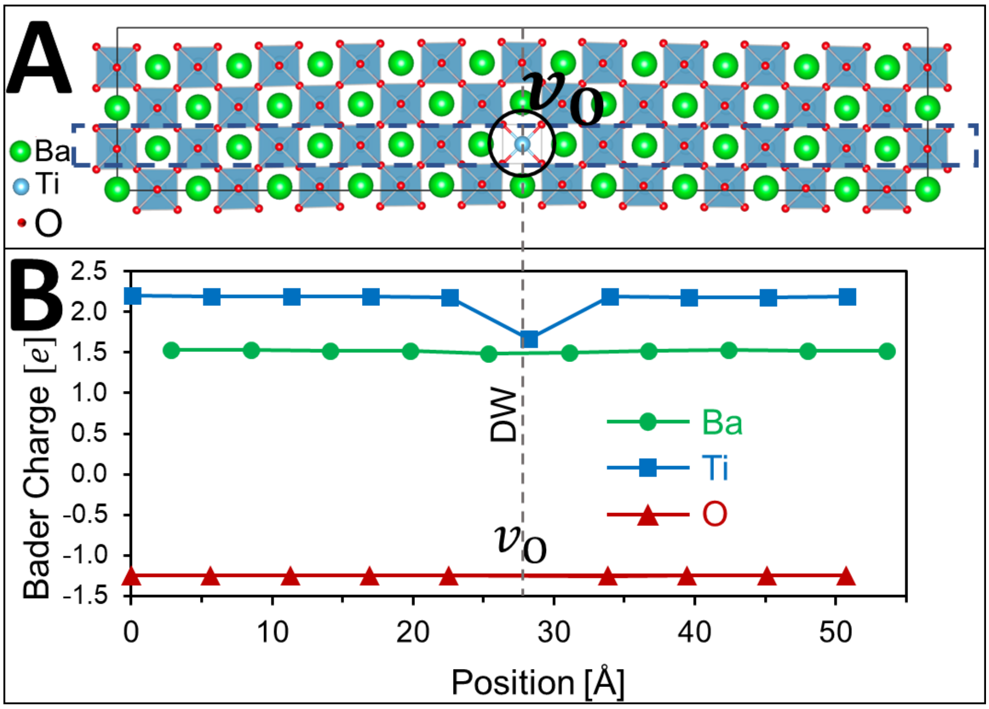

Figure S8| Reduced Ti ion at the oxygen vacancy site, observed by Bader charge calculation.

Density of States (DOS) for $PbTiO_3$

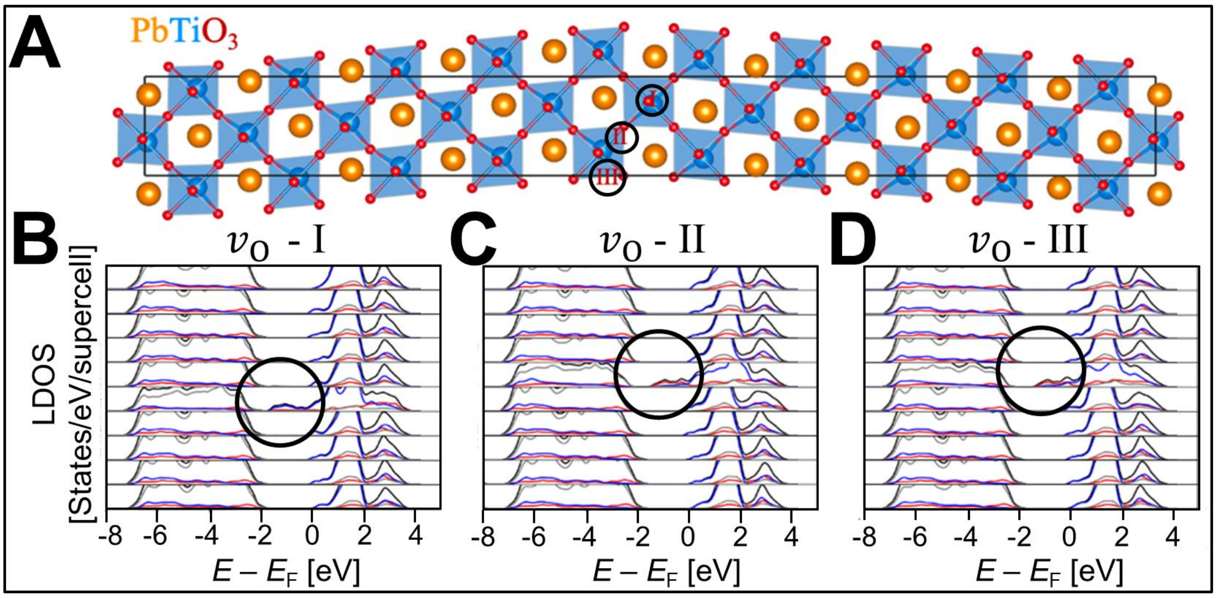

Figure S9| DOS for $PbTiO_3$ with three different vacancies. (**A**) The unit cell arrangement of $PbTiO_3$. (**B-D**) DOS for three different vacant sites, showing additional states in all sites, supporting the proposal of local conductivity. Vacant I and III are equatorial sites, while vacant II is the axial site in reference to the polarization direction.

Oxygen-vacancy formation at an existing domain wall

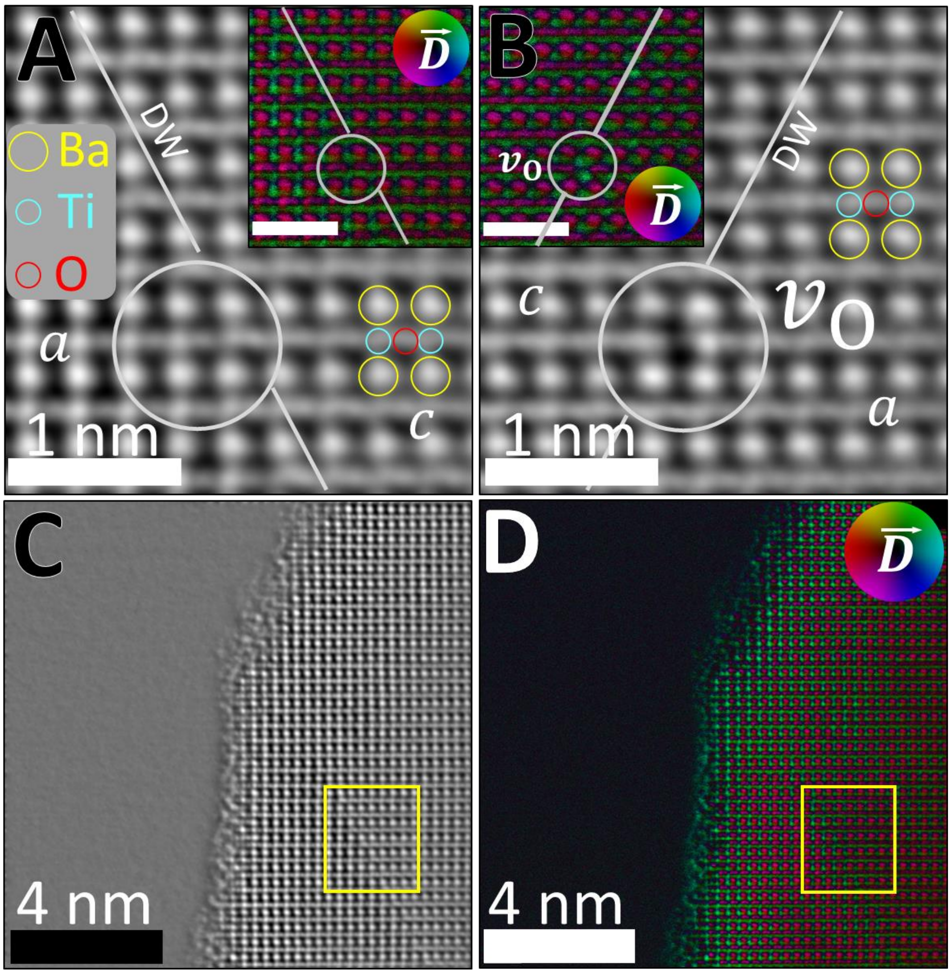

Figure S10| Oxygen-vacancy formation at a switched domain wall. (**A**) iDPC (insert: DPC) scan of neighboring *a* and *c* domains with a domain wall (designated) in between that contains no v_O. (**B**) The same area after a long exposure (1290 nA/nm^2) that resulted in domain-wall rotation (designated) and oxygen-vacancy formation at the point where the previous and rotated domain walls intersect. Yellow boxes denote the region of interest. Color wheel in (D) represents the orientation (color) and intensity (hue) of the electric-field displacement vector.

References

(1) Barzilay, M.; Ivry, Y. Formation and Manipulation of Domain Walls with 2 nm Domain Periodicity in $BaTiO_3$ without Contact Electrodes. *Nanoscale* **2020**, *12* (20), 11136–11142.

(2) Meyer, B.; Vanderbilt, D. *Ab Initio* Study of Ferroelectric Domain Walls in $PbTiO_3$. *Phys. Rev. B* **2002**, *65*, 104111.

(3) Kwei, G. H.; Lawson, A. C.; Billinge, S. J. L.; Cheong, S. W. Structures of the Ferroelectric Phases of Barium Titanate. *J. Phys. Chem.* **1993**, *97* (10), 2368-2377.

(4) He, L.; Vanderbilt, D. First-Principles Study of Oxygen-Vacancy Pinning of Domain Walls in $PbTiO_3$. *Phys. Rev. B* **2003**, *68*, 134103.

(5) Chandrasekaran, A.; Damjanovic, D.; Setter, N.; Marzari, N. Defect Ordering and Defect-Domain-Wall Interactions in $PbTiO_3$: A First-Principles Study. *Phys. Rev. B* **2013**, *88*, 214116.

(6) Wang, X.; Xu, T.; Xuan, F.; Chen, C.; Shimada, T.; Kitamura, T. Effect of the Oxygen Vacancy on the Ferroelectricity of 90° Domain Wall Structure in $PbTiO_3$: A Density Functional Theory Study. *J. Appl. Phys.* **2019**, *126* (17), 174107.

(7) Costa, -A. R.; Gohda, Y. Role of Ferroelectricity, Delocalization, and Occupancy of *d* States in the Electrical Control of Interface-Induced Magnetization. *Phys. Rev. Appl.* **2021**, *15* (6), 064014.
